# Associations between human leukocyte antigens and renal function

**DOI:** 10.1038/s41598-021-82361-7

**Published:** 2021-02-04

**Authors:** Marcus Lowe, Antony Payton, Arpana Verma, Judith Worthington, Isla Gemmell, Patrick Hamilton, William Ollier, Titus Augustine, Kay Poulton

**Affiliations:** 1 Transplantation Laboratory, Manchester Royal Infirmary, Manchester University NHS Foundation Trust, Manchester, UK; 2 Division of Informatics, Imaging and Data Sciences, School of Health Sciences, Faculty of Biology, Medicine and Health, University of Manchester, Manchester, UK; 3 Division of Population Health, Health Services Research and Primary Care, University of Manchester, Manchester, UK; 4 Department of Renal and Pancreatic Transplantation, Manchester Royal Infirmary, Manchester University NHS Foundation Trust, Manchester, UK; 5 Centre for Bioscience, Faculty of Science and Engineering, Manchester Metropolitan University, Manchester, UK; 6 Division of Diabetes, Endocrinology and Gastroenterology, University of Manchester, Manchester, UK

**Keywords:** Kidney, Genetic association study, Immunogenetics

## Abstract

Human leukocyte antigens (HLA) have been associated with renal function, but previous studies report contradictory findings with little consensus on the exact nature or impact of this observation. This study included 401,307 white British subjects aged 39–73 when they were recruited by UK Biobank. Subjects’ HLA types were imputed using HLA*IMP:02 software. Regression analysis was used to compare 362 imputed HLA types with estimated glomerular filtration rate (eGFR) as a primary outcome and clinical indications as secondary outcome measures. 22 imputed HLA types were associated with increased eGFR (and therefore increased renal function). Decreased eGFR (decreased renal function) was associated with 11 imputed HLA types, seven of which were also associated with increased risk of end-stage renal disease and/or chronic kidney disease. Many of these HLA types are commonly inherited together in established haplotypes, for example: HLA-A*01:01, B*08:01, C*07:01, DRB1*03:01, DQB1*02:01. This haplotype has a population frequency of 9.5% in England and each allele was associated with decreased renal function. 33 imputed HLA types were associated with kidney function in white British subjects. Linkage disequilibrium in HLA heritance suggests that this is not random and particularly affects carriers of established haplotypes. This could have important applications for the diagnosis and treatment of renal disease and global population health.

## Introduction

Approximately 1.2 million people died due to chronic kidney disease (CKD) worldwide in 2015^1^, representing an increase of 32% since 2005. CKD is now ranked 17th in the list of diseases which cause the most “years of lost life”, rising from 21st in 2005 and 25th in 1990^1^. 2.6 million people received dialysis in 2010, and treatment of end-stage renal disease (ESRD) accounts for 2–3% of the healthcare budgets of high-income countries^2^. Understanding the genetic influences which predispose people to kidney dysfunction will have important applications for the diagnosis and treatment of a globally significant disease.

A number of human leukocyte antigens (HLA) encoded within the major histocompatibility complex are associated with increased or decreased risk of renal failure^3^. For example, HLA-B*51 was associated with ESRD in Venezuelan^4^ and Brazilian^5^ subjects, while A*26 was protective against ESRD in Saudi Arabia^6^ and Turkey^7^. Approximately 100 different HLA types have been linked to renal function by various studies worldwide^8^, including studies of European or white populations^9–13^. These were mostly reported in case–control studies comprising fewer than 500 subjects of a single nationality, and many associations remain unreplicated or have been contradicted by other studies. Despite mounting evidence that an association between HLA and renal function exists, there is no supportive confirmation in sufficiently-powered studies. We interrogated a large cohort of white British subjects to test the hypothesis that the HLA region is associated with renal function.

## Methods

### Study population and quality control

This is a UK Biobank (UKB) retrospective cohort study using data from 502,616 subjects aged 39–73 years at the time of recruitment between 2006 and 2010^14^. 88% of the cohort self-identifies as “white British”, and principal component analysis conducted by UKB concluded that 82% of the UKB cohort is white British^15^. Analysis was restricted to this group to reduce population stratification; 92,858 subjects who were not white British were analysed separately^16^.

Individuals within the cohort whom UKB deemed to be related^17^ (kinship coefficient ≥ 0.044) were also excluded (n = 7318) to avoid HLA frequency bias^18^. Where subjects were related, the individual with the most complete set of genetic data, based on a set of “high-quality markers”^17^, was included. Genetic sex influences kidney function^19^, so only individuals whose sex could be clearly assigned were included. Subjects identified by UKB to have sex chromosome karyotypes other than XX or XY^20^ and those whose genetic sex, as calculated by UKB, did not match their self-reported sex^21^ were removed (n = 786 in total). Finally, 347 subjects were excluded at UKB’s recommendation due to missing genetic data^22^. A total of 101,309 subjects were excluded during quality control, leaving 401,307 subjects for analysis. All quality control was performed using Stata/SE 13.0 (StataCorp).

### HLA typing

Imputation estimates a person’s most likely HLA type based on the presence of particular single nucleotide polymorphisms^23^. HLA types were imputed for each subject by UKB using HLA*IMP:02 software^24^ at the following loci: HLA-A, B, C (Class I) and DPA1, DPB1, DQA1, DQB1, DRB1, DRB3, DRB4, DRB5 (Class II)^25^ at a level equivalent to high resolution typing using eight reference datasets^26^. 362 HLA types were imputed. Two of these (HLA-DQB1*02:02 and DPB1*03:01) were not in Hardy–Weinberg equilibrium (HWE, P < 0.00014) so were excluded from this study; the remaining 360 alleles were included. Table 1 shows the 100 HLA types with frequency > 1% in the cohort.

**Table 1:** HLA types with frequency > 1%. This shows the 44 Class I and 56 Class II types which have frequency > 1% in the cohort. They are split into Class I and Class II and sorted in descending order of frequency.

Class I		Class II	
HLA−	Frequency (%)	HLA−	Frequency (%)
A*02:01	27.44	DPB1*04:01	43.55
A*01:01	19.42	DRB4—no gene	35.02
C*07:01	17.64	DRB3—no gene	34.33
C*07:02	15.82	DRB4*01:03	25.35
B*07:02	14.82	DQA1*05:01	23.00
A*03:01	14.48	DQA1*03:01	20.35
B*08:01	14.44	DQA1*01:02	19.09
C*05:01	11.38	DPA1*01:03	18.44
B*44:02	11.20	DQB1*03:01	17.53
C*06:02	8.97	DRB3*01:01	16.74
C*04:01	8.26	DRB5—no gene	15.27
C*03:04	7.97	DQB1*02:01	14.94
A*24:02	7.25	DRB1*03:01	14.87
B*15:01	6.11	DPA1*02:01	14.60
A*11:01	6.04	DQA1*02:01	14.58
B*44:03	5.85	DRB1*07:01	14.56
B*40:01	5.56	DRB1*15:01	14.47
C*03:03	5.54	DRB5*01:01	14.44
B*35:01	4.52	DQB1*06:02	14.19
C*16:01	4.43	DQA1*01:01	14.14
A*29:02	4.24	DRB3*02:02	13.24
B*27:05	3.95	DQB1*05:01	12.00
B*57:01	3.92	DRB1*04:01	11.14
C*08:02	3.62	DPB1*02:01	10.76
B*51:01	3.60	DPB1*04:02	10.69
B*18:01	3.59	DQB1*03:02	10.37
C*02:02	3.59	DRB1*01:01	9.33
A*32:01	3.47	DQB1*02:02a	9.27
C*01:02	3.41	DPB1*03:01a	9.11
A*68:01	3.01	DRB4*01:01	8.06
C*12:03	2.82	DPB1*01:01	5.92
A*31:01	2.68	DQA1*01:03	5.44
B*14:02	2.52	DQB1*03:03	5.23
B*13:02	1.93	DQB1*06:03	5.06
A*26:01	1.85	DRB1*13:01	5.00
B*55:01	1.83	DRB1*04:04	3.98
C*07:04	1.76	DRB1*13:02	3.79
C*15:02	1.73	DRB3*03:01	3.77
A*23:01	1.70	DRB1*11:01	3.19
A*25:01	1.62	DPA1*02:02	3.10
B*37:01	1.33	DQB1*06:04	2.74
B*14:01	1.16	DPB1*11:01	2.60
B*49:01	1.15	DQB1*05:03	2.17
A*30:01	1.01	DPB1*05:01	2.07
		DQB1*04:02	2.00
		DQA1*04:01	1.95
		DRB1*14:01	1.91
		DRB1*08:01	1.79
		DRB1*01:03	1.69
		DPB1*10:01	1.61
		DPB1*13:01	1.49
		DRB1*12:01	1.42
		DRB1*09:01	1.31
		DPB1*17:01	1.11
		DRB1*11:04	1.04
		DQB1*06:09	1.02

^a^Not in Hardy–Weinberg equilibrium so excluded from analysis.

### Measures of renal function

Renal function was determined using estimated glomerular filtration rate (eGFR), a measure of toxin filtration calculated using serum biomarkers such as creatinine and cystatin^27^. High levels of these biomarkers are indicative of poor renal function and manifest in a lower eGFR. This study used the Chronic Kidney Disease Epidemiology Collaboration (CKD-EPI) eGFR calculation which adjusted for age and sex^28^. Three eGFR values were calculated for each subject, using measures of: creatinine; cystatin; and both creatinine and cystatin^29^. Pairwise correlation confirmed that the three eGFR values were similar (Pearson’s correlation coefficients > 0.6; P < 0.0001). The cystatin-based eGFR value provided the most complete dataset; only this value was used for analysis to avoid repetition of testing using closely correlated variables.

Clinical histories for each subject were used as secondary outcomes. Subjects with kidney dysfunction were identified by examining self-reported questionnaires in addition to data relating to clinical diagnosis and procedures undertaken. These were deduced using a combination of the International Classification of Diseases^30^ (ICD)-9 and -10, Office of Population Censuses and Surveys (OPCS)^31^-3 and -4, and UKB’s own coding systems^32,33^. Subjects were categorised as: ESRD patients (yes or no); kidney transplant recipients (yes or no); dependent on renal replacement therapy (RRT) including transplantation at any point (yes or no); and CKD patients of any stage (yes or no) (see Table 6).

### Statistical analysis

Linear regression analysis was performed to test for associations between HLA alleles and eGFR as a continuous variable. All 360 HLA alleles which were in HWE were included, with a Bonferroni threshold of P < 0.00014 considered significant^34^ (0.05/360). Subjects who had ever received RRT were excluded as their eGFR values may have suggested healthy renal function even though their native function was poor.

Logistic regression was used to test for associations between HLA types and adverse clinical outcomes (ESRD, RRT, CKD, and kidney transplantation; binary variables). Age at recruitment and sex were included as covariates, and only alleles in HWE with minor allele frequency > 5% were considered (n = 50) in order to increase statistical power. P < 0.001 was considered significant after Bonferroni correction. All regression analysis was performed using Plink software^35^.

### Ethical approval

All methods were carried out in accordance with relevant guidelines and regulations. All experimental protocols were approved by UKB’s Research Ethics Committee. Informed consent was obtained for all subjects. UKB has obtained Research Tissue Bank approval from its ethics committee that covers the majority of proposed uses of the resource, so researchers do not typically need to obtain separate ethics approval.

## Results

### Variation in renal function

Variation in renal function within the UKB cohort is outlined in Table 2. eGFR values could not be calculated for around 18,000 subjects (< 5%) due to missing creatinine and/or cystatin measurements. Subjects dependent on RRT were excluded from the analysis, although their eGFR values are listed in Table 2, which shows calculated eGFR values and the corresponding CKD stages^36^ as well as the number of subjects in the final analysis.

**Table 2:** Distribution of subjects’ eGFR.

Subjects with eGFR	CKD stage	Kidney function determined by		
		Creatinine	Cystatin	Both
0–14.9	5 (ESRD requiring RRT)	95	91	92
15–29.9	4	277	538	374
30–44.9	3b	1147	2611	1436
45–59.9	3a	7164	14,777	7575
60–89.9	2 (when combined with other evidence of kidney damage)	148,885	183,525	172,037
90–119.9	1 (when combined with other evidence of kidney damage)	225,142	180,139	199,325
≥ 120		694	1877	2287
Data missing		17,903	17,749	18,181
Excluded due to RRT		1357	1354	1351
Included in eGFR analysis		382,047	382,204	381,775

This shows the distribution of subjects’ eGFRs for each of the three eGFR formulae, as well as the number of subjects included and excluded. eGFR thresholds are based on thresholds used for CKD diagnosis. Some subjects were dependent on RRT so were not included in analysis of eGFR, though their eGFRs are included in this table.

The calculated eGFR values were compared to average values to check that they were plausible. Average eGFR for different age categories were taken from the National Kidney Foundation^37^. The values calculated by this study were in line with NKF’s estimates, as shown in Table 3. This increases confidence in the calculated values.

**Table 3:** eGFR values by age.

Age	Average eGFR according to			
	National Kidney Foundation	This study (creatinine)	This study (cystatin)	This study (both)
40 s	99	99.6	100.0	100.7
50 s	93	92.4	90.2	92.1
60 s	85	85.0	80.5	83.5

This shows average eGFR values by decade of life for the general population (provided by National Kidney Foundation) and for the three eGFR formulae used in this study.

11,379 subjects were identified with ESRD. Of these, 437 were renal transplant recipients and 1412 subjects had RRT. 4794 subjects had a clinical diagnosis of CKD (36 stage 1, 300 stage 2, 3557 stage 3, 397 stage 4, and 504 stage 5).

### Regression analysis

33 HLA types were significantly associated with renal function after correction for multiple testing. Table 4 lists the 11 HLA alleles linked with decreased renal function (defined by either decreased eGFR or the presence of CKD or ESRD). Table 5 shows the 22 HLA alleles associated with increased renal function. No HLA associations were identified with kidney transplant status or RRT status. Tables 4 and 5 also show the population frequency of the alleles, the beta value or odds ratio (OR) of each effect, and the level of significance of the associations.

**Table 4:** Alleles which are associated with decreased kidney function.

HLA-	Frequency (%)	eGFR based on cystatin		CKD		ESRD	
		Beta	P	OR	P	OR	P
A*01:01	19.4	− 0.26	9.98E−09			1.062	0.00033
A*03:01	14.5	− 0.22	2.65E−05				
B*07:02	14.8	− 0.20	8.84E−05				
B*08:01	14.4	− 0.48	3.26E−20	1.109	0.00027	1.066	0.00072
C*07:01	17.6	− 0.35	1.68E−13	1.098	0.00038		
C*07:02	15.8	− 0.21	2.35E−05				
DQA1*05:01	23.0	− 0.38	2.03E−18	1.105	3.30E−05		
DQB1*02:01	14.9	− 0.49	7.06E−22	1.121	4.43E−05	1.084	1.44E−05
DRB1*03:01	14.9	− 0.49	1.14E−21	1.122	3.61E−05	1.077	6.33E−05
DRB3—no gene	34.3	− 0.31	6.09E−16	1.090	6.35E−05		
DRB3*01:01	16.7	− 0.42	7.57E−18				

This shows the alleles significantly associated with decreased kidney function as well as the frequency of the alleles, the beta value or odds ratio (OR) of each effect, and the P values.

**Table 5:** Alleles which are associated with increased kidney function.

HLA-	Frequency (%)	eGFR based on cystatin	
		Beta	P
A*25:01	1.6	0.77	1.07E−07
A*29:02	4.2	0.39	1.37E−05
A*32:01	3.5	0.38	0.00014
B*14:01	1.2	0.67	8.62E−05
B*14:02	2.5	0.64	3.43E−08
B*44:03	5.9	0.34	1.31E−05
C*12:03	2.8	0.63	7.99E−09
C*16:01	4.4	0.40	5.79E−06
C*02:02	3.6	0.39	8.64E−05
C*05:01	11.4	0.23	4.57E−05
C*08:02	3.6	0.63	1.04E−10
DQA1*02:01	14.6	0.24	4.23E−06
DQA1*03:01	20.4	0.31	8.12E−12
DQB1*03:02	10.4	0.29	1.60E−06
DQB1*06:01	0.4	1.15	4.63E−05
DQB1*06:09	1.0	0.72	7.39E−05
DRB1*15:02	0.4	1.10	9.85E−05
DRB1*04:01	11.1	0.30	3.23E−07
DRB1*07:01	14.6	0.22	1.46E−05
DRB4—no gene	35.0	0.33	5.64E−18
DRB4*01:01	8.1	0.38	1.43E−08
DRB4*01:03	25.4	0.23	5.85E−08

This shows the alleles significantly associated with increased kidney function as well as the frequency of the alleles, the beta value of each effect, and the P values.

### Associations with decreased renal function

HLA types are inherited in maternal and paternal haplotypes and are not randomly distributed. Of the 11 HLA associations with decreased eGFR, seven were also linked to development of CKD, ESRD or both. 10 of these 11 HLA alleles are inherited in two well-documented haplotypes: (HLA-A*01:01, B*08:01, C*07:01, DRB1*03:01, DRB3*01:01, DQA1*05:01, DQB1*02:01; and A*03:01, B*07:02, C*07:02^38^. All genes in the former are associated with decreased eGFR, and all but HLA-DRB3*01:01 are also linked to increased risk of CKD, ESRD, or both. This haplotype is seen in 9.5% of the English population^39^. The “absence of DRB3 genes” was also associated with decreased eGFR, which may either indicate increased susceptibility in subjects homozygous for this common haplotype, or may reflect individuals with the latter haplotype, which is in linkage disequilibrium (LD) with HLA-DRB1*15:01 and therefore has no associated DRB3 genes present. Alternatively, a closely linked haplotype, HLA-A*03:01, B*07:02, C*07:02, DRB1*03:01, DQB1*02:01 (present in 0.5% of the English population^39^) may be implicated here.

### Associations with increased renal function

The HLA associations with increased eGFR values do not appear to belong to full length haplotypes, but can be separated into groups of two or three HLA alleles which are often co-inherited. For example: HLA-DRB1*04:01, DQA1*03:01, DQB1*03:02 (seen in 8.2% of the English population^40^); DRB1*07:01, DQA1*02:01 (10.5% of the English population^40^); A*29:02, B*14:02, C*08:02 (2.1% of the Northern Irish population^40^); and B*44:03, C*16:01 (4.7% of the Northern Irish population^40^, also commonly associated with A*25:01) were all linked to increased eGFR. None of the 22 alleles associated with increased eGFR was shown to reduce the risk of adverse renal-related clinical outcomes.

## Discussion

We identified significant HLA associations with renal function in the largest reported study to date. 22 HLA alleles were associated with increased renal function and 11 with decreased function. The HLA associations with increased renal function did not suggest a protective effect against CKD or ESRD, but the 11 associations with decreased renal function (seven of which were also linked to ESRD and/or CKD) were of particular interest. HLA genes are inherited through maternal and paternal haplotypes, which suggests a high probability that these alleles are not independently associated with renal function, but rather that this observation is non-random within the population. Specifically, individuals who carry the haplotypes listed are at increased risk of developing renal dysfunction, and may carry sub-clinical levels of impairment even in the absence of identifiable disease. This clustering of the HLA genes within well-documented haplotypes adds validity, which is reinforced as the primary and secondary outcome measures were calculated using the independent phenotypes of biomarkers and clinical outcomes. It should be noted that some significant alleles appear to be alone in significance (that is, the alleles that they are in LD with were not significant). Examples include HLA-A*32:01 and B*14:01, among others. In these cases, it is possible that the allele itself is linked to kidney function, independent of its haplotype, or it is possible that the other alleles in LD with this allele are also significant, and this study failed to detect this. The CKD-EPI calculation of eGFR was selected rather than MDRD^41^ or Cockcroft-Gault^42^ due to its increased accuracy when assessing subjects with normal renal function (eGFR > 60)^43^. Using only one eGFR value avoided multiple testing of closely related variables; the formula based on cystatin was selected as it had the fewest missing values. For comparison, the two other CKD-EPI eGFR formulae (one based on creatinine, and another based on both creatinine and cystatin) were used and the data re-analysed. In addition to the associations already described, three additional associations were identified as significant (assuming the same Bonferroni threshold of P < 0.00014): HLA-A*23:01 and DRB3*02:02 were linked to decreased renal function, and B*27:05 was linked to increased function.

### Comparison with previous research

Previous literature has reported conflicting HLA associations with renal function in populations of different ethnic origin. Potentially, these contradictory findings may include false positives arising from inadequate statistical power, multiple testing, publication bias or methodological differences. Alternatively, it is possible that HLA associations with kidney function differ between populations due to varied heritage. Limiting this study to only white British subjects reduced any likelihood of bias due to population stratification.

Almost 100 HLA associations with ESRD have been described, only 11 of which have been confirmed by two or more independent studies. Our study replicated one of these 11 observations but refuted two. HLA-DRB1*03 was previously associated with renal dysfunction by four groups with a combined total of 1261 ESRD subjects and over 3000 controls^5–7,44^. We found not only HLA-DRB1*03:01 but an entire haplotype to be associated with decreased eGFR and increased risk of poor clinical outcome. However, HLA-B*07 was reported to be protective against ESRD in 1620 ESRD patients and 1211 controls by Doxiadis et al.^10^, and Karahan’s study of 587 patients and 2643 controls^7^. In this population, HLA-B*07:02 was associated with decreased renal function. Furthermore, HLA-DRB1*04 was associated with adverse renal outcomes in three previous studies with over 4000 ESRD subjects^12,45,46^, but here, DRB1*04:01 was linked to increased renal function. The remaining eight previously replicated HLA associations were not significant in this study. Overall, 14 of our associations confirmed previous observations^5–7,12,44,45,47–49^, while 12 of our findings refuted previous results^7,10,12,45,46,49,50^.

It is worth noting, however, that this study is much larger than any previous study. Most previous studies used case–control methodology (see “Strengths and weaknesses” below) and many failed adjust for multiple testing. Therefore, the findings reported here, which have undergone more stringent statistical testing, may be less prone to type I or II error.

### Implications

This study is unique in that some of the HLA alleles associated with decreased renal function form a well-characterised haplotype. Both this and individual component HLA alleles have been associated with multiple diseases which result in CKD or ESRD, including systemic lupus erythematosus and IgA deficiency^51^. Our study indicates that even within a healthy population, renal function may be sub-clinically impaired in subjects with these alleles. These findings have the potential to impact upon clinical practice. HLA typing is already used as a diagnostic tool for disorders with strong HLA associations such as coeliac disease^52^, ankylosing spondylitis^53^, and actinic prurigo^54^. It may be advisable for clinicians to use HLA disease association typing to aid the diagnosis of renal failure, which could ensure timely therapeutic intervention. However, HLA associations with these diseases are much stronger than those reported here: the association between B*27 and ankylosing spondylitis has an odds ratio of 171^55^, while HLA associations with coeliac disease have OR > 10^56^, compared to ORs < 1.13 in this study. Clinicians and national kidney transplantation programmes may also use the HLA types associated with increased renal function to help identify suitable kidney donors.

### Strengths and weaknesses

A key advantage of this study is the cohort size, which is larger than any previously published research. 382,204 subjects were included in the analysis of the primary outcome measure (eGFR), and the secondary analysis consisted of 11,379 cases of ESRD (and 389,928 controls). This study uses a variety of measures of renal function, most of which are calculated independently and are therefore unlikely to be subject to systemic bias. eGFR is a useful outcome measure because it provides a continuous scale, giving an accurate and precise estimate of renal function. Many previous studies used case–control methodology, reducing kidney function from a spectrum to binary categorisations such as “ESRD or healthy”. Measuring renal function on a spectrum may strengthen the statistical and clinical significance of this study.

A limitation of this investigation is that the HLA typing was performed by imputation rather than direct genotyping, which is more accurate^57^. This is because the cost of HLA typing a cohort of this size using traditional methods is prohibitively expensive. The imputation program used for the UKB population was HLA*IMP:02, though Karnes’ review^57^ of competing programs suggests that SNP2HLA is more accurate. Nevertheless, the review stated that HLA*IMP:02 is 94% accurate when imputing white subjects which, given the size of our cohort, is acceptable within the scope of this study. Furthermore, 360 of the 362 imputed alleles were in HWE (P > 0.00014), suggesting that the majority of imputed allele frequencies were consistent with frequencies that might be expected in a stable population. The two alleles which were not in HWE (HLA-DQB1*02:02 and DPB1*03:01) were excluded from the analysis.

Some HLA associations found in this study do not appear to be part of a haplotype. These alleles may be independently associated with renal function, or they may be false positives caused by inaccurate imputation. For example, HLA-B*44:02 was not significant in this study, but is commonly associated with C*05:01, DRB1*04:01, DQA1*03:01 and DQB1*03:02 in combination with either A*25:01, A*29:02, or A*32:01, which were all linked to increased renal function. Our study identified HLA-B*44:03, rather than B*44:02, to be associated with increased renal function. This discrepancy may suggest that HLA-B*44:02 alleles were incorrectly imputed as B*44:03, though given that C*16:01 (which is often seen in LD with B*44:03) was also associated with increased renal function may validate the observation regarding B*44:03. Any dubious observations may be resolved by repeating the imputation using an alternative imputation programme or additional reference panels.

It is possible that the strategy employed to identify subjects with adverse kidney-related clinical outcomes was insufficiently comprehensive to capture all cases. If data held by UKB were incomplete, or if relevant codes were not included (see Table 6), subjects with poor renal outcomes would be mischaracterised as healthy. This could be averted by obtaining a peer-reviewed validation of the coding systems that documents exactly which codes are representative of adverse renal outcomes, but to the best of our knowledge no such validation exists. Clinical outcomes were secondary outcome measures in this study; the primary outcome of eGFR is not affected by this limitation.

**Table 6:** Codes included in definitions of ESRD, kidney transplant status, RRT status, and CKD. This shows the various coding systems and codes used to identify subjects with adverse clinical outcomes.

Coding system	Code	Decoded	ESRD patient	RRT recipient	Kidney transplant recipient	CKD patient
Coding 5	1195	Renal/kidney transplant	Yes	Yes	Yes	
	1476	Fistula for dialysis	Yes	Yes		
	1580	Dialysis access surgery	Yes	Yes		
	1581	Haemodialysis access/fistula surgery	Yes	Yes		
	1582	Peritoneal dialysis (CAPD) access surgery	Yes	Yes		
Coding 6	1192	Renal/kidney failure	Yes			
	1193	Renal failure requiring dialysis	Yes	Yes		
	1194	Renal failure not requiring dialysis	Yes			
ICD-10	I120	Hypertensive renal disease with renal failure	Yes			
	I131	Hypertensive heart and renal disease with renal failure	Yes			
	I132	Hypertensive heart and renal disease with both (congestive) heart failure and renal failure	Yes			
	N17	Acute renal failure	Yes			
	N170	Acute renal failure with tubular necrosis	Yes			
	N172	Acute renal failure with medullary necrosis	Yes			
	N178	Other acute renal failure	Yes			
	N179	Acute renal failure, unspecified	Yes			
	N18	Chronic renal failure	Yes			
	N180	End-stage renal disease	Yes			
	N181	Chronic kidney disease, stage 1				Yes
	N182	Chronic kidney disease, stage 2				Yes
	N183	Chronic kidney disease, stage 3				Yes
	N184	Chronic kidney disease, stage 4				Yes
	N185	Chronic kidney disease, stage 5	Yes			Yes
	N188	Other chronic renal failure	Yes			
	N19	Unspecified renal failure	Yes			
	P960	Congenital renal failure	Yes			
	T861	Kidney transplant failure and rejection	Yes	Yes	Yes	
	Y841	Kidney dialysis	Yes	Yes		
	Z49	Care involving dialysis	Yes	Yes		
	Z491	Extracorporeal dialysis	Yes	Yes		
	Z492	Other dialysis	Yes	Yes		
	Z940	Kidney transplant status	Yes	Yes	Yes	
	Z992	Dependence on renal dialysis	Yes	Yes		
ICD-9	584	Acute renal failure	Yes			
	585	Chronic renal failure	Yes			
	586	Renal failure, unspecified	Yes			
	5845	Acute renal failure with lesion of tubular necrosis	Yes			
	5846	Acute renal failure with lesion of renal cortical necrosis	Yes			
	5847	Acute renal failure with lesion of renal medullary (papillary) necrosis	Yes			
	5848	Acute renal failure with other specified pathological lesion in kidney	Yes			
	5849	Acute renal failure, unspecified	Yes			
	5859	Chronic renal failure	Yes			
	5869	Renal failure, unspecified	Yes			
	77980	Congenital renal failure	Yes			
	E8791	Abn. reaction to kidney dialysis without misadventure at time	Yes	Yes		
	V451	Renal dialysis status	Yes	Yes		
	V56	Aftercare involving intermittent dialysis	Yes	Yes		
	V560	Aftercare involving extracorporeal dialysis	Yes	Yes		
	V568	Aftercare involving other dialysis	Yes	Yes		
N/A	<15	eGFR based on creatinine	Yes			
	<15	eGFR based on cystatin	Yes			
	<15	eGFR based on both creatinine and cystatin	Yes			
OPCS3	566	Transplantation of kidney	Yes	Yes	Yes	
	4013	Paracentesis abdomini: peritoneal dialysis	Yes	Yes		
	4695	Other operations on intestine, not elsewhere classified: isolation loop for dialysis	Yes	Yes		
	5661	Transplantation of kidney: donor	Yes	Yes	Yes	
	5662	Transplantation of kidney: cadaver	Yes	Yes	Yes	
	9503	Other vascular procedures: haemodialysis	Yes	Yes		
OPCS4	L746	Creation of graft fistula for dialysis	Yes	Yes		
	M01	Transplantation of kidney	Yes	Yes	Yes	
	M011	Autotransplantation of kidney	Yes	Yes	Yes	
	M012	Allotransplantation of kidney from live donor	Yes	Yes	Yes	
	M013	Allotransplantation of kidney from cadaver NEC	Yes	Yes	Yes	
	M014	Allotransplantation of kidney from cadaver heart beating	Yes	Yes	Yes	
	M015	Allotransplantation of kidney from cadaver heart non-beating	Yes	Yes	Yes	
	M018	Other specified transplantation of kidney	Yes	Yes	Yes	
	M019	Unspecified transplantation of kidney	Yes	Yes	Yes	
	M172	Pre-transplantation of kidney work-up—recipient	Yes	Yes	Yes	
	M174	Post-transplantation of kidney examination—recipient	Yes	Yes	Yes	
	X40	Compensation for renal failure	Yes	Yes		
	X401	Renal dialysis	Yes	Yes		
	X402	Peritoneal dialysis NEC	Yes	Yes		
	X403	Haemodialysis NEC	Yes	Yes		
	X404	Haemofiltration	Yes	Yes		
	X405	Automated peritoneal dialysis	Yes	Yes		
	X406	Continuous ambulatory peritoneal dialysis	Yes	Yes		
	X407	Haemoperfusion	Yes	Yes		
	X408	Other specified compensation for renal failure	Yes	Yes		
	X409	Unspecified compensation for renal failure	Yes	Yes		
	X41	Placement of ambulatory apparatus for compensation for renal failure	Yes	Yes		
	X411	Insertion of ambulatory peritoneal dialysis catheter	Yes	Yes		
	X412	Removal of ambulatory peritoneal dialysis catheter	Yes	Yes		
	X418	Other specified placement of ambulatory apparatus for compensation for renal failure	Yes	Yes		
	X419	Unspecified placement of ambulatory apparatus for compensation for renal failure	Yes	Yes		
	X42	Placement of other apparatus for compensation for renal failure	Yes	Yes		
	X421	Insertion of temporary peritoneal dialysis catheter	Yes	Yes		
	X428	Other specified placement of other apparatus for compensation for renal failure	Yes	Yes		
	X429	Unspecified placement of other apparatus for compensation for renal failure	Yes	Yes		
	X431	Extracorporeal albumin haemodialysis	Yes	Yes		
UKB data	42026	End stage renal disease report	Yes			
	42027	Date of end stage renal disease report	Yes			

A final limitation of this study is that the sizes of the associations with eGFR were smaller than previously published HLA disease associations^55,56^ and possibly too small to be considered clinically relevant. 25 out of 33 (76%) significant associations with eGFR had a beta value between − 0.5 and 0.5, suggesting that the presence of the allele has only a minor effect on kidney function. However, in seven cases, these apparently small effects were corroborated by associations with adverse clinical outcomes, implying that small beta values are not a contraindication of clinical relevance.

## Conclusions

This study has identified 22 HLA types which are associated with increased kidney function, and 11 which are linked to decreased kidney function in a large UK population. Many of these are commonly inherited together in haplotypes. Importantly, seven alleles, which are each seen in between 14–34% of the cohort, were linked to both decreased eGFR and increased incidence of adverse clinical outcomes. Due to the constitution of the cohort, the results of this study can only be applied to white British people aged 39–73. Repeating the analyses with alternative cohorts may add considerably to our current knowledge and allow a better assessment on the implications for population health.
